# Fiber Loop Ringdown Sensor for Potential Real-Time Monitoring of Cracks in Concrete Structures: An Exploratory Study

**DOI:** 10.3390/s130100039

**Published:** 2012-12-20

**Authors:** Peeyush Sahay, Malik Kaya, Chuji Wang

**Affiliations:** Department of Physics and Astronomy, Mississippi State University, Starkville, MS 39762, USA; E-Mails: ps329@msstate.edu (P.S.); mk400@msstate.edu (M.K.)

**Keywords:** fiber loop ringdown, crack sensors, concrete

## Abstract

A fiber loop ringdown (FLRD) concrete crack sensor is described for the first time. A bare single mode fiber (SMF), without using other optical components or chemical coatings, *etc.*, was utilized to construct the sensor head, which was driven by a FLRD sensor system. The performance of the sensor was evaluated on concrete bars with dimensions 20 cm × 5 cm × 5 cm, made in our laboratory. Cracks were produced manually and the responses of the sensor were recorded in terms of ringdown times. The sensor demonstrated detection of the surface crack width (SCW) of 0.5 mm, which leads to a theoretical SCW detection limit of 31 μm. The sensor's response to a cracking event is near real-time (1.5 s). A large dynamic range of crack detection ranging from a few microns (μm) to a few millimeters is expected from this sensor. With the distinct features, such as simplicity, temperature independence, near real-time response, high SCW detection sensitivity, and a large dynamic range, this FLRD crack sensor appears promising for detections of cracks when embedded in concrete.

## 1. Introduction

Health monitoring of concrete structures, including crack monitoring, is an important requirement in the civil infrastructures [1]. Apart from the causes, such as natural hazards, earthquakes, *etc.*, other factors responsible for cracks in concrete structures are aging, thermal contraction upon drying, shrinkage due to water unbalance, sub-grade settlements, applied loads, *etc.* [2]. Depending on the location, cracks may or may not be visible. A crack on the surface of a structure is easily detectable, whereas cracks inside a structure may not be apparent at all. Similarly, depending on the extent and location of cracks, damage severity to the structure can be different. For example, a crack width of 0.3 mm is sufficient to allow water penetration inside concrete blocks which consequently can result in corrosion. Likewise, even a micro-crack at critical points, such as joints, bending, *etc.*, can be extremely dangerous and requires immediate care. Crack monitoring, therefore is an essential part of structural health monitoring (SHM).

There are various non-destructive techniques for sensing cracks in concrete structures, for example, the surface penetrating radar method, impact-eco method, infrared thermography, acoustic emissions, *etc.* [3–6]. In addition, in recent years, a new technology called smart aggregate that uses embedded piezoceramic based transducers has also been used to monitor cracks in concrete structures [7–10]. More details on the conventional techniques involved in crack sensing can be found elsewhere [11,12]. With regard to SHM, the first use of optical fiber sensors is generally credited to Mèndez *et al.* [13]. Compared to the conventional techniques of sensing cracks in concrete structures, techniques based on optical fiber sensing have their own advantages. For example, fiber optic sensors (FOS) are immune to electromagnetic interferences, functional in harsh environments, of small footprint, and low-cost [14,15]. Based on sensing mechanism, FOS can be categorized as: intensiometric sensors, interferometric sensors, fiber Bragg grating (FBG) sensors, and polarimetric sensors [16]. All of these sensors have their respective merits and limitations. For instance, intensiometric sensors are capable of long range sensing with the simplest sensing mechanism; whereas interferometric sensors, FBG sensors, and polarimetric sensors are useful in localized sensing, and they involve complex instrumentation [17]. Similarly, on the one hand, performance of intensiometric sensors is affected by light fluctuations [18]; the FBG based sensors are affected by temperature fluctuations and they require use of additional means to counter the temperature impact [19]. A detailed discussion on different FOS regarding their applications, performances, advantages, limitations, *etc.*, in view of concrete health monitoring can be seen in several excellent reviews [16,17,20–26].

Among the aforementioned FOS, the intensiometric sensors, which use intensity modulation for measurements, are the simplest to construct. In principle, they are capable of sensing an event along the whole length of the optical fiber cable; therefore they can detect damages or cracks at any point in the concrete along the fiber line. In one of the earliest works involving concrete damage detection using the intensity modulation technique, Rossi and Le Maou [27] conducted experiments with a bare fiber for crack detection in concrete structures. The fiber, with its protective coatings removed, was embedded directly in the concrete, and the transmitted signal was monitored. As the crack reached to the fiber, the fiber broke, causing abrupt cessation of the transmitting signal. Although the simplest, the major limitation of this method is that once the fiber breaks no further detection can be performed. Ansari and Navalurkar [28] designed their sensors for crack detection based on the same intensity modulation method yet with a different configuration. To increase the sensitivity, the fiber was made in a loop shape such that the fiber circumferences the generated crack. The sensor based on this design is limited to small size cracks only. Leung *et al.* [29] developed a sensor to monitor flexural cracks in the concrete structures. The loss in the back scattered light intensity is related to a mechanical deformation. The arrangement of the fiber which is laid in a zig-zag course inside the concrete is the key feature of this design. This design increases the sensitivity of the system. The sensor is efficient in monitoring flexural cracks under various types of loads. This technique is simple and sensitive, but only responsive to certain orientations of cracks with regard to the fiber's orientation. Habel *et al.* [30] demonstrated that an intensity-based FOS can be used in a quasi-distributed configuration to measure crack opening widths. Similarly, Lee *et al.* [31] showed that even a low resolution and less sensitive intensity based optical fiber sensor constructed with inexpensive instruments can be useful in the cases where precise measurements of strain or cracks are not required, for example, measurements of stiffness.

In general, for health monitoring of concrete structures, including damage detection, an ideal technique should have the common desirables: a simple sensing mechanism, a long sensing range, low instrumentation cost, high sensitivity, fast response, insensitive to temperature and light fluctuations, and capability of distributed sensing [32]. In the present work, we describe a new fiber loop ringdown (FLRD) sensor, which potentially meets the aforementioned requirements for crack detection in concrete structures.

The FLRD technique originates from cavity ringdown spectroscopy (CRDS). In CRDS, a light pulse is injected into a cavity constructed using two highly reflective mirrors. The trapped light pulse bounces back and forth many times before it dies out completely. In each round trip a small part of the light energy of the trapped light pulse leaks out of the cavity. The temporal profile of this transmitted light intensity exhibits a single exponential decay. The decay rate of the light intensity generates the sensing signal—“ringdown time”, from which, concentration of a gas inside the cavity can be determined [33–35]. Involving from the principle of CRDS, the FLRD technique utilizes the decrease rate of the light intensity in a closed fiber loop to determine the ringdown time. The ringdown time changes on account of different optical losses of the light pulse traveling inside the fiber loop. The difference in the ringdown time results from a change in the optical loss, which is related to a sensing event occurred in one section (sensor head) of the fiber loop. The FLRD technique was first demonstrated by Stewart *et al.* [36]. Later many different variants of FLRD have been reported by different research groups for different applications [37–43], including pressure, force, and strain sensors using a fiber loop combined with different types of fibers or optical components, such as FBG and long period grating [44–48]. However, to date the FLRD technique has not been explored for crack detection in concrete structures.

Of various FLRD-based sensors, this is the first FLRD-based crack sensor that is fabricated, packaged, and embedded in concrete for testing. Highly sensitive and temperature-independent FLRD crack sensors have been developed to monitor cracks in concrete slabs. A bare single mode fiber (SMF) was used as a sensor head, which picks up a sensing event, a cracking event in this case. The sensors were tested in our laboratory with actual concrete bars. Sensors were embedded in a wet concrete slab, so that upon drying out of the concrete, the sensor was integrated with the concrete slab and became one unit. Cracks were produced manually; the responses of the sensors to the produced cracks were monitored as a change in the ringdown time. Crack detection sensitivity in terms of surface crack width (SCW) of the concrete slab on the order of tens micron (μm) was estimated theoretically. Although, the conventional FOS, such as FBG, Fabry-Perot sensors, Brillouin based sensors, *etc.*, can have a strain sensitivity as high as 0.1 με [16] or can detect a crack of size as small as sub-millimeters [49], they all involve complicated instrumentation. Given the simplicity and low instrument cost, the present FLRD crack sensor may represent a new type of crack sensor in SHM.

## 2. Sensor Design and Sensing Principle

This section first describes the experimental setup for the FLRD sensors and then explains the sensing principle of the technique.

### 2.1. FLRD Sensors

**Figure 1.**
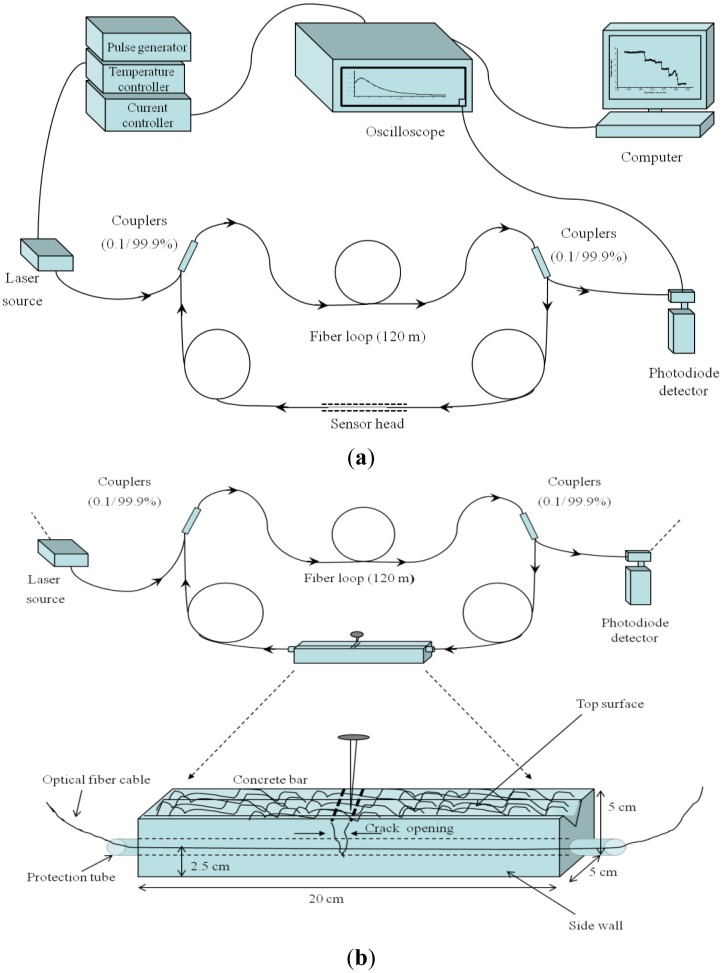
(**a**) Schematic of the FLRD sensor system. (**b**) Sensor configuration for crack sensing in concrete bar.

A typical FLRD sensor system for crack detection is depicted in Figure 1. A FLRD sensor system consists of two major sections: a FLRD sensor unit and its control system. The FLRD sensor unit was constructed with a SMF loop (SMF-28e, Corning Inc., Painted Post, NY, USA) that was formed through two identical 2 × 1 fiber couplers (Opneti Communication Co., Hong Kong); in the middle of the 120 m long fiber loop, one small segment, *i.e.*, 1–20 cm, of the bare fiber was chosen to serve as the sensor head. No modification or special treatment was needed to construct the sensor head; instead the small segment of the bare fiber was used as it is for this purpose. The main components of the FLRD sensor control system include a continuous wave (cw) diode laser (NTT electronics), laser control electronics, a photodiode detector (PDA50B, Thorlabs, Newton, NJ, USA.), and a ringdown data acquisition portion. The control system used in this work was the same as the ones described elsewhere [40,42]. In general, a FLRD sensor unit, with different sensing functions, can be controlled by the same sensor control system. The connection and disconnection of a fiber sensor unit to the control system was readily achieved via two SMF FC/APC connectors.

SMF, having a tensile stress ≥ 100 kspi, a fatigue parameter N_d_ = 20, and diameters of the cladding and core being 125 μm and ∼8.2 μm, respectively, was used to construct the 120 m long loop. The split ratio at the two-leg end was 0.1:99.9. The connection of the fiber couplers to the fiber loop is as shown in Figure 1. Optical losses of the light in the fiber loop are absorption losses, fiber connectors' insertion losses, and fiber couplers' losses. A total loss of <0.45 dB was estimated for each fiber loop fabricated in the present study. Ringdown signals were detected by the photodiode detector. A detected signal was fed to a pulse generator to produce a series of negative square waves. These pulsed square waves were applied to the laser driver to drop the laser current to zero rapidly; consequently a series of laser pulses from the continuous wave diode laser were created. A detailed description of a FLRD sensor system can be seen elsewhere [40,42,50].

### 2.2. FLRD Sensing Principle

A light pulse when coupled into a fiber loop makes many round trips inside the loop. Intensity of the light pulse decreases in each round trip because of the internal optical loss. The photodiode detector observes different intensities of the transmitted light from each round trip. Therefore, the rate of change of the light intensity as observed by the detector can be given as [39],

(1) $$\frac{\text{dI}}{\text{dt}}=-\frac{\text{IAc}}{\text{nL}}$$

where *I* is the light intensity at any arbitrary time *t*, *A* is the total fiber transmission loss of the light per round trip; *c* is the speed of the light. *n* and *L* represent the average refractive index and the total length of the fiber loop, respectively. The temporal behavior of the light intensity *I* can be obtained from Equation (2):

(2) $$I=I_{0}e^{‒\frac{c}{\text{nL}}\text{At}}$$

The time it takes for the intensity to decrease from *I_o_* to *I_o_*/*e* is termed as the ringdown time, *τ*_0_, and is given by Equation (3a):

(3a) $$\tau_{0}=\frac{\text{nL}}{\text{cA}}$$

(3b) $$\tau =\frac{\text{nL}}{c(A+B)}$$

For a given FLRD sensor, the total transmission loss *A* depends on the physical parameters of the sensor, such as the fiber absorption loss, the couplers' insertion losses, the refractive index, and the fiber length. Typically, for a given fiber loop, *A* remains constant. The term *B* represents the additional optical loss of the light pulse which occurs as a result of a sensing activity at any section of the fiber loop (*i.e.*, sensor head). This causes a change in the ringdown time, *τ*, given by Equation (3b). From Equations (3a) and (3b), we have:

(4) $$B=\frac{\text{nL}}{c}(\frac{1}{\tau }-\frac{1}{\tau_{0}})$$

Equation (4) shows that an additional optical loss, *B*, can be determined by measuring the two ringdown times *τ* and *τ_0_*. Therefore, Equation (4), suggests that a change resulting from a sensing activity, such as external pressure, deformation, absorption, *etc.*, can be determined by measuring ringdown times with and without the sensing event. Earlier, FLRD was demonstrated for pressure or force sensing due to micro-bending [39,40]. In this work, the FLRD technique is further explored to detect cracking events in concrete structures. We first investigated the stretching characteristics of the single mode fiber (elongation in length) to understand the limit of SMF stretching; thereafter experiments were conducted for crack sensing in concrete bars.

Assume that a small portion of the fiber in the middle of the fiber loop is stretched by a small length Δ*L*, if *α* is the loss per unit stretch length, the total loss due to the stretch Δ*L* occurring in the small portion of the fiber loop, can be given as *α*Δ*L*. Therefore, Equation (3a) is modified to:

(5) $$\tau =\frac{n(L+\Delta L)}{c(A+\alpha \Delta L)}$$

In the case of small stretches, *i.e.*, when Δ*L* is on the order of millimeters against the length of fiber loops of several meters, *i.e.*, 120 m in the present case, we can safely assume: *L* + Δ*L* ≈ *L*, therefore:

(6) $$\tau =\frac{\text{nL}}{c(A+\alpha \Delta L)}$$

If the loss due to the stretching is considerably smaller than the total optical loss in the fiber loop, *i.e.*, αΔL ≪ A, then:

(7a) $$\tau =\frac{\text{nL}}{\text{cA}}{\left(1+\frac{\alpha \Delta L}{A}\right)}^{-1}$$

(7b) $$\tau =\tau_{0}\left(1+\frac{\alpha }{A}\Delta L\right)$$

Equation (7b) exhibits a linear relationship between the ringdown time and the stretched length. The ringdown time, *τ*, is directly proportional to the decrease in the stretched length, Δ*L*, in the fiber.

**Figure 2.**
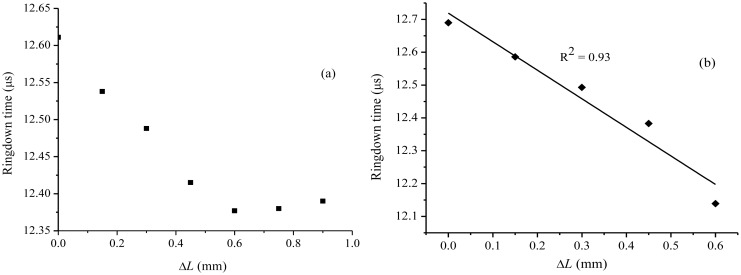
(**a**) A graph of the ringdown time *versus* the stretched length (Δ*L*) from the fiber stretch experiment; upto the breaking threshold of the fiber. (**b**) Result from the repeated fiber stretch experiment.

Two sets of experiments were conducted to examine the relation expressed in Equation (7). Two points were marked in a small section of the optical fiber in the middle of the loop. One of the marked parts was glued to a fixed mount, and the other marked part was glued to a mount attached to a high precision translation platform with a spatial resolution of ±10 μm. With one mount fixed, the other was moved horizontally to create a stretch in the fiber. Stretches were developed in steps; the ringdown time, *τ*, was recorded each time when the stretch length was increased. A graph of *τ versus* Δ*L* is plotted in Figure 2.

The experiment was conducted with a section of fiber of 8 cm long. The stretches in the fiber were produced in steps. The ringdown time first decreased with increase in the stretched length. A decrease of 0.23 μs in *τ* was recorded for the Δ*L* = 0.6 mm. Fitting the experimental curve to a line yielded R^2^ = 0.98, which showed that the decrement in the ringdown time was fairly linear in this range. However, for Δ*L* > 0.60 mm, the ringdown time was noted to increase. The ringdown time, 12.37 μs at Δ*L* = 0.60 mm, increased to 12.38 and 12.39 μs, at Δ*L* = 0.75 and 0.90 mm, respectively. The fiber was broken when further stretched. This suggested that the section of 8 cm long SMF had a tolerance level (the breaking point) of 0.9 mm. The experiment was repeated. A similar graph, *τ versus* Δ*L*, was plotted for this repeated experiment is shown in the Figure 2(b). For an increase in the stretched length in the fiber from 0 to 0.6 mm, the ringdown time decreased from 12.69 to 12.13 μs. A linearity of R^2^ = 0.93 was obtained. The fiber was not stretched further in order to avoid its breaking. The part showing non-linear response of the fiber beyond a particular Δ*L*, 0.6 mm in this case, is attributed to the fact that in a stretched optical fiber cable, beyond a certain limit of the stretched length in the fiber, the field propagating inside the fiber cable does not remain to be confined in the fiber core. The non-linearity arises as a result of coupling differences between the higher order excitations in the cladding part of the optical fiber and the lower order excitations in the fiber core. The similar phenomenon was also reported in an early work [51].

The experimental results validated the relationship between *τ* and Δ*L*, as derived in Equation (7). These stretch characterization results suggest that a bare single mode fiber can be utilized to investigate fiber stretch resulting from structure deformation, including cracks. Furthermore, for a given section of SMF of 8 cm long, the maximum stretch length can be up to 0.6 mm. If the stretch is fully due to a structure separation resulting from a crack, the width of crack-opening can also be determined. This is the research hypothesis to be studied in this work.

### 2.3. Concrete Samples

**Figure 3.**
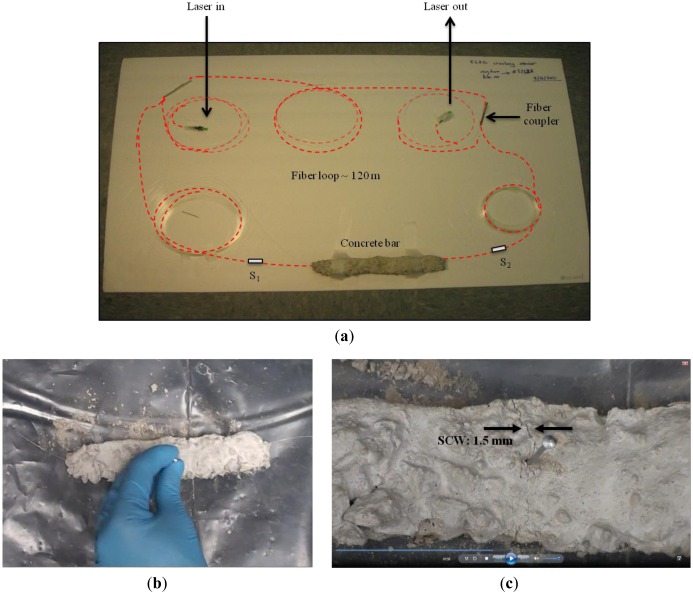
(**a**) An image of a FLRD crack sensing unit: showing the fiber loop connected to the sensor head (concrete bar). (**b**) An image showing the manual procedures to produce cracks in the concrete bar. (**c**) A typical surface crack as it appears on the top surface of the bar.

Rectangular bar-shaped concrete units were prepared manually by mixing the ready-to-use concrete mix (Quikrete, Atlanta, GA, USA) and water with a mix ratio of 3:1. The dimensions of the bars were approximately 20 cm × 5 cm × 5 cm (length × width × height). The wet concrete was poured into a box, made up of cardboard that later on was removed after the concrete dried out. While curing and drying out of a concrete bar, where a section of bare SMF was laid down, the section of the fiber remain embedded inside the concrete bar, making an integrated sensor unit. It would be worth mentioning that the section of the optical fiber that was laid down in the concrete was a bare SMF cable without any treatment or modification. Two flexible rubber tubes however were used at the two ends of the concrete bar to prevent the optical fiber from being cut by the sharp edges at the corner of the bar. A typical FLRD crack sensor unit is shown in Figure 3. The fiber was laid down along the longest symmetry axis of the rectangular bar without stretch. The perpendicular distance from the fiber to the surface of bar is about 2.5 cm. In the similar manner, relatively softer grout bars were made by adding tile mortar with polymer (Mapei, Deerfield Beach, FL, USA) to the concrete mixture. Both the concrete bars and the grout bars took approximately two days to settle and dry out.

Three sensor units, namely *units-1*, *2*, and *3*, were fabricated. The *unit-1* was made of the ready concrete mix; *unit-2* and *unit-3* were made of the concrete and grout mixtures. Each bar unit had one section of bare SMF embedded. Approximately 15 cm long fiber cable was extended outside the bar at each end through a flexible rubber tube as a protection means. Once a bar dried out, the sections of the fibers extended outside the bars at the two ends were spliced to form a fiber loop, as shown in Figure 3(a). Characteristics of the concrete and grout bars, *i.e.*, *units-1*, *2*, and *3*, are discussed in the later section. One single ringdown loop was utilized to test the three sensor units individually.

### 2.4. A Sensor Unit

The picture in Figure 3(a) shows a FLRD sensor unit constructed for the experiment. The concrete bar was spliced to the fiber loop through the junctions *S_1_* and *S_2_*, as shown in the figure. A laser pulse was injected into the loop through the FC/APC connector on the input arm of the fiber loop. The output arm of the loop was connected to a photodiode detector. The ringdown decay waveform was monitored by an oscilloscope which was connected to a laptop computer for data processing.

Figure 3(b) shows the method of producing cracks in the concrete bars: a nail, 6d size (2 inches), was manually hammered into the bar. The nail was hit gently in steps till the crack started appearing on the surface of the bar. With the nail positioned at the same place further hitting increased the crack width on the surface, called surface crack width (SCW). Figure 3(c) shows the actual image of a typical surface crack. The crack line is almost normal to the fiber line.

**Figure 4.**
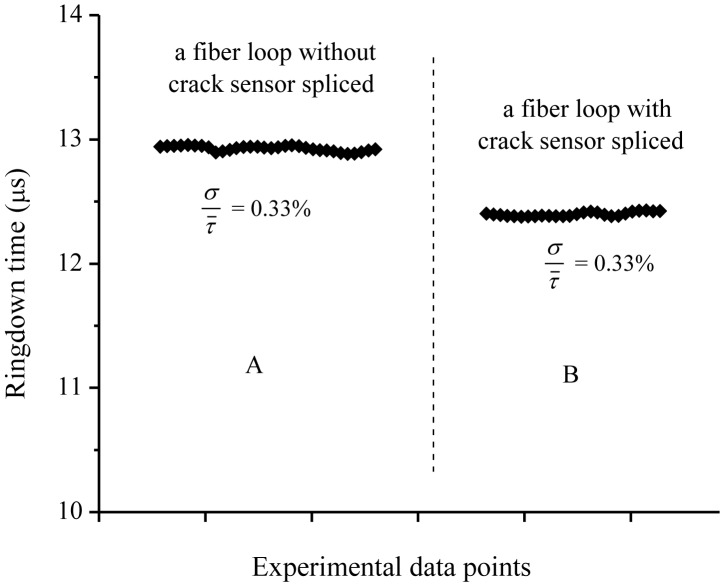
Typical sensor baseline stabilities (**A**) without (**B**) with a sensor head spliced into the fiber loop. Both have the same baseline stability of 0.33% while their baselines are different due to different total losses.

In order to check the signal stability, the ringdown baseline stability which is defined as *σ*/*τ̅*, where *σ* is the standard deviation and *τ̅* is the base ringdown time, was determined [40]. The baseline stability was determined to be 0.33% by averaging over 100 ringdown events for both the cases, namely, the fiber loop without a crack sensor head integrated and the fiber loop with a crack sensor head (the bar units) connected. Figure 4 shows a comparison of the baseline stabilities in the two cases. The part *A* in Figure 4 represents the ringdown data collected when the sensor was not attached to the loop. The ringdown time (the baseline, *τ*_0_) in this case was ∼12.8 μs with a baseline stability of 0.33%. The part *B* represents the ringdown data when the sensor was connected (spliced) to the loop. The ringdown time in this case was 12.4 μs, with the same baseline stability, 0.33%. These results suggested that the signal in the fiber loop was quite stable and the splicing process (integrating a sensor head into the fiber to form a loop) did not generate additional noise to the sensor's signal. A lower ringdown time in the case of the fiber loop spliced with the concrete bar unit is due to the additional optical losses occurring at the two splicing junctions.

## 3. Results and Discussion

### 3.1. Response of FLRD Crack Sensors

**Figure 5.**
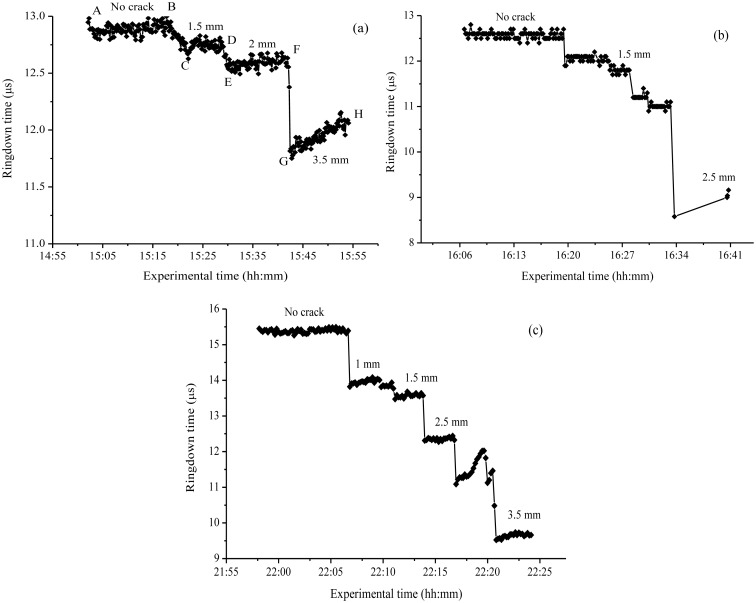
Responses of FLRD crack sensors to different surface crack widths; Figure 5(**a**–**c**), are the responses of *units-1*, *2*, and *3*, respectively.

Figure 5 shows the results from the crack sensing experiments. A cracking event created on the surface of the bar generated a stress on the fiber embedded in the bar, thus observed ringdown time decreased due to additional optical loss resulting from the fiber stress. The sensor's response to the cracking event was near real-time (∼1.5 s). The ringdown time, τ, averaged over 100 ringdown events, was recorded at different crack widths (SCW) that were produced in steps. In accordance with the sensing principle expressed in Equation (7b), the ringdown time was noted to decrease with increase in SCW. Experiments were conducted with all of the three units. Results of the experiments are shown in Figure 5(a–c), for the *units-1*, *2*, and *3*, respectively.

It should be noted that the crack width against which the ringdown time has been plotted in Figure 5 is the crack width measured on the top surface of the bars, the SCW. As depicted in Figure 1(b), the crack first appeared on the surface, which upon further hitting propagated down inside the concrete bar. Sensing of the cracking event was realized by observing a decrease in the ringdown time; thereafter, upon every hitting a proportional decrease in *τ* was recorded. It would be worth mentioning here that the crack appearing on the surface, viewed either from the top or the side wall, does not necessarily means that the same amplitude of crack is generated at the location where the fiber is embedded. In this work, there was no exact mechanism or method to estimate the actual crack width at the location of optical fiber embedded. The only physically measureable quantity was SCW. That is why SCW has been used to plot against the ringdown time in order to examine the response of the sensor system in crack detection.

Owing to the heterogeneous distribution of the constituents in the concrete bar, the crack was less likely to propagate uniformly inside the bar. Nevertheless, the result shown in Figure 5 supports that the fiber embedded inside the concrete did sense the cracking events generated on top surface of the concrete bar. Moreover, a step-wise decrease in the ringdown time indicates the increasing cracking effect at the fiber location. Figure 5(a) shows the response of the sensor *unit-1* to a set of three SCWs. From point *A* to point *B* is the ringdown times recorded with the sensor without cracking events created. At the point *B*, the nail was started to be hammered slowly till a substantial change in the ringdown time could be observed on the computer screen. The point *C* was marked when the ringdown time was 12.7 μs. The slant part from *B* to *C* represents the time elapsed before the first considerable change in the ringdown time was noticed, which in turn indicated about the propagation of the cracking effect that happened from the surface of the bar to the optical fiber location. The concrete bar at this point of time had developed a few additional surface cracks as well. At this stage, a SCW of 1.5 mm along the width of the bar was measured; the data were recorded from the points *C* to *D*. The ringdown time during this time period remained to be approximately 12.7 μs. At the point *D*, the bar was hammered again. It resulted in a sharp decrease in ringdown time, reaching the point *E* with τ = 12.5 μs. At the same time, cracks in other directions also widened. The data were recorded for the time period from *E* to *F*, with SCW had increased to 2.0 mm. At the point *F*, the bar was hammered once again; there was a huge drop in the ringdown time, reaching τ = 11.8 μs. SCW at this point was 3.5 mm; and the cracks in other directions widened much more; and the bar was at the brink of breaking. When the sensor was left for few minutes, the ringdown time slowly increased back to 12 μs. This behavior is speculated to be due to the post-hit relaxation of the fiber inside the bar.

**Figure 6.**
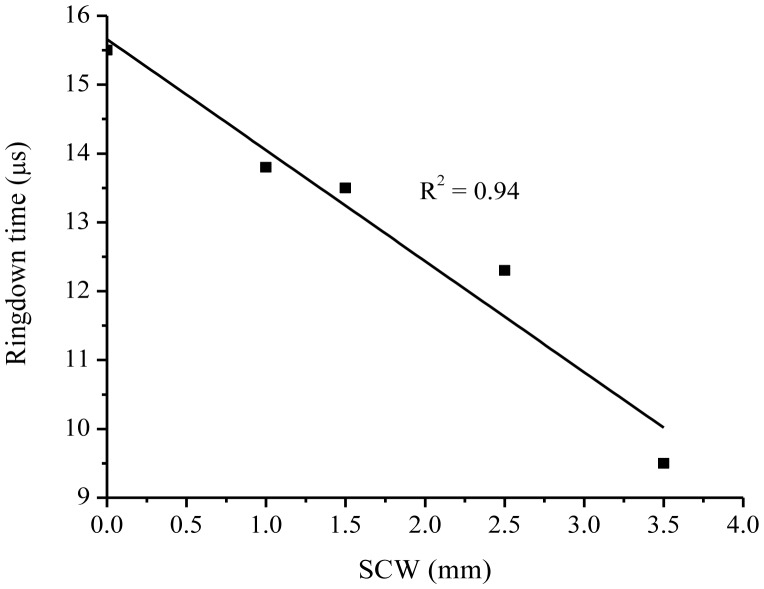
Response of the sensor *unit-3* exhibiting a linear relation between the ringdown time and the SCW.

The *unit-1*, made up of the concrete-mix and water only, was hard in structure. Production of uniform cracks in a controlled manner could not be achieved by manually hitting the nail. Therefore, it was desirable to make better concrete bars to handle cracks. With this concern, two additional sensors, *unit-2* and *unit-3*, were created by adding grout mortar-mix (Mapei) to the concrete mixture, so that the bars were relatively softer. The compositions of *unit-2* and *unit-3* were mixtures of concrete-mix:grout:water in a ratio of 3:3:2, respectively. These bars allowed production of cracks in a more controlled manner. Experiments were repeated. Figure 5(b) shows the response of *unit-2*. A decrease in ringdown time from 12.5 μs to 9.0 μs was observed from no cracking in the concrete bar to a SCW of 2.5 mm. A sharp decrease in ringdown time, shown by the vertical drop lines in the figure, corresponding to a responding time of 1.5 s, indicates the fast response of the sensor. The ringdown signal remained stable and consistent every time when SCW increased. The results of *unit-3*, obtained in the similar way, are shown in Figure 5(c). A SCW up to 3.5 mm was produced; with the sensor responding to an every single cracking event on the surface of the bar. Significant drops in ringdown time were recorded for an each cracking event. Cracks produced in steps with SCW of 1, 1.5, 2.5, and 3.5 mm, resulted in ringdown times of 13.8, 13.5, 12.3, and 9.5 μs, respectively. The larger change in ringdown time for the same change in SCW of 3.5 mm, as compared to the *unit-1*, indicated that this sensor unit had a better sensitivity. A plot of ringdown time, τ, *versus* SCW measured for *unit-3* is shown in Figure 6. Although, a quantitative relation between the ringdown time and the SCW cannot be predicted beforehand, the linear decrease in ringdown time with increase in the SCW suggests that the cracking events produced on the surface generated linearly proportional stresses to the optical fiber embedded inside in the bar. Therefore, from the results we can conclude that a cracking event occurring at the surface of the bar, 2.5 cm above the sensor head can be detected and the cracking amplitude is reflected by the change in ringdown time. It must be stressed that this type of cracking sensor is better to be used for continuous crack monitoring instead of measurement of crack widths inside a concrete structure. A calibration curve, obtained in a computer-simulated and controlled cracking situation, may be helpful in determining the widths of actual crack-openings inside the concrete; and this is a subject of future work.

### 3.2. Detection Sensitivity of Surface Crack Width

As mentioned earlier, each of the three sensor units, *unit-1*, *2*, and *3*, in terms of crack productions, responded differently to the cracking events. Therefore, in order to examine the SCW detection sensitivity of the sensors units, the results obtained need to be looked at individually.

In the case of *unit-1*, a decrease of 0.2 μs in ringdown time was observed when the SCW increased from 0 (no crack) to 1.5 mm. However, the next cracking event increased the SCW to 2 mm; an increment of 0.5 mm. An equal decrease of 0.2 μs in ringdown time was recorded. This suggested that once the crack on the surface propagated down to the optical fiber location, the sensor exhibited a SCW detection sensitivity of 0.5 mm.

Similar were the observations for sensor *unit-2* and *unit-3*. Experimentally, the difference among the three sensor units was the production of controlled cracks. Unlike in *unit-1*, relatively controlled cracks were produced in the sensor *units-2* and *3*; and both sensor units responded promptly to an every cracking event generated on the top surface of the bar. A uniform step-wise increase of 0.5 mm in SCW was achieved with both the sensor units, as shown in Figure 5(b,c). A substantial decrease in ringdown time in each increasing step in SCW was noted with near real-time response (1.5 s). Conservatively, both sensor units can be considered to be sensitive to a SCW of 0.5 mm or smaller. This estimation is based on the fact that the production of a SCW smaller than 0.5 mm in each step could not be achieved and controlled in the present experimental situation. However, considerable changes in ringdown time for a SCW of 0.5 mm in each step, as shown in Figures 5(b,c), indicate that the sensor could theoretically be much more sensitive in terms of response to a much smaller SCW. This speculation drives a further consideration of a theoretical detection sensitivity of sensors in terms of a minimum detectable SCW (described in the section below).

### 3.3. Theoretical Detection Sensitivity of the Crack Sensor

The theoretical detection sensitivity of FLRD crack sensors can be estimated by using the baseline stability of the ringdown signals. The baseline stability, *σ*/*τ̅*, (expressed in %) is interpreted as the minimum fractional ringdown time that comes from a minimum distinguishable *τ* from two separate signals under a given set of experimental conditions. That means, two signals, say *τ*_1_ and *τ*_2_, can be distinguished only if the difference between them is equal to at least one-σ (the one-σ standard deviation). From Equation (7b), we derive:

(8a) $$\Delta \tau =\tau_{0}-\tau =\tau_{0}\frac{\alpha }{A}\Delta L$$

(8b) Δτ=mΔL

The Δ*τ* in Equation (8) represents the decrease in the ringdown time, (τ_0_ > τ), with the increase in fiber stretched length Δ*L*; $m=\tau_{0}\frac{\alpha }{A}$, is the slope of the line in the graph of Δ*τ versus* Δ*L*. The slope *m* is determined experimentally.

It should be noted that Δ*L* in Equation (8) is the actual stretched length of the fiber; whereas the only physically measurable quantity in this experiment is SCW. However, as discussed earlier, Δ*L* is proportionally related to SCW, therefore Equation (8) must hold true for SCW as well. Therefore, rewriting Equation (8) for SCWs, Δ*d*, we have:

(9) Δτ=mΔd

**Figure 7.**
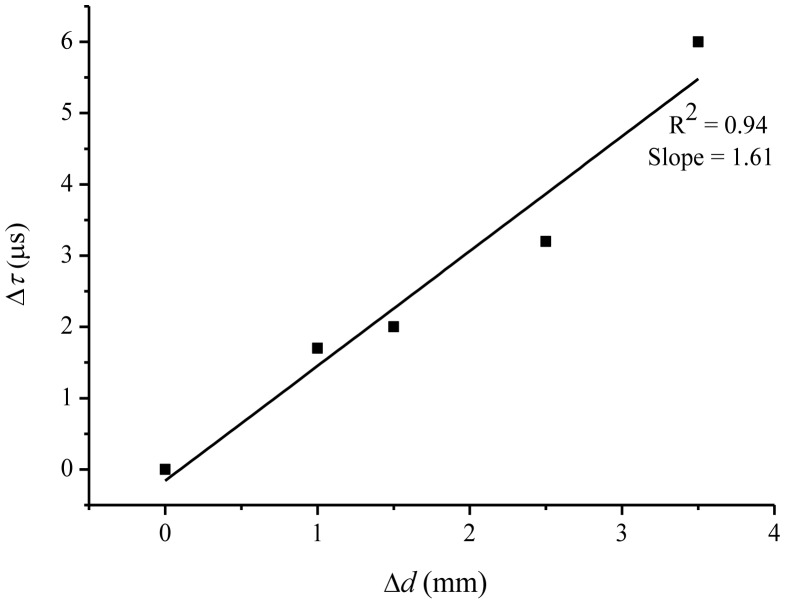
A calibration curve of the decreased ringdown time (Δ*τ*) *vs.* SCW (Δ*d*), obtained from the sensor *unit-3*.

Further, from Equation (9), it can be derived that:

(10) $$\Delta d_{\min}=\frac{1}{m}\Delta \tau_{\min}$$

where Δ*d*_min_ is the minimum measurable SCW; $\Delta \tau_{\min}=\left(\frac{\sigma }{\bar{\tau }}\right)\tau_{0}$, the minimum measurable ringdown time which can be determined with a known baseline stability and a ringdown baseline. A graph between Δ*τ* and Δ*d*, based on the experimental results obtained for the *unit-3*, is plotted in Figure 7. The graph attains linearity of R^2^ = 0. 94 and a slope *m* = 1.61.

On the other hand, using the one-σ standard deviation, Δ*τ*_min_ of 0.0511 μs, is determined for the baseline stability, 0.33%, and the ringdown baseline, 15.50 μs. Consequently, a minimum measurable SCW, Δ*d*_min_ = 31 μm, was determined.

This implies that, theoretically, the presented FLRD crack sensor is responsive to a surface crack width as small as 31 μm, in particular for the sensor *unit-3*. This study suggests that although the actual crack widths at the fiber location may not be determined at this stage, a cracking event happening on the surface of a concrete structure can certainly be sensed by the sensor, with a theoretical detection sensitivity of microns. A detailed investigation into the detection sensitivity requires experiments be carried out under controlled conditions.

### 3.4. Advantages and Limitations of the FLRD Crack Sensors

The FLRD crack sensor has several unique advantages in comparison to its counterparts: (i) simplicity, (ii) temperature independence, (iii) near real-time response, and (iv) high detection sensitivity and large dynamic range:

- Simplicity: The presented FLRD crack sensors offer simplicity in terms of construction and operation. A bare single mode fiber, without using any advanced fiber optic components or chemical coatings, is directly utilized as a sensor head for the purpose of sensing. Consequently, the use of SMF offers ease of construction as well as low cost of embedment in concrete structures, unlike other conventional sensors based on FBG, Brillouin scattering, or Fabry–Perot techniques, which involve complicated instrumentation procedures and special cares in the sensor embedment [23,49,52]. Furthermore, the FLRD crack sensor uses an inexpensive photodiode as the detector, significantly reducing costs in the terminal detection equipment.
- Temperature independence: The FLRD crack sensor is based on strain sensing mechanism. Due to the low thermal coefficient, 0.5 × 10^−6^ °C, of the silica fiber [40,53] and free of other optical components in the sensor head, the FLRD crack sensor is virtually independent of environmental temperature in the range of −169–800 °C [54]. This type of crack sensor is especially advantageous when temperature variations are an important factor, *i.e.*, in combustion facility, reactors, *etc.*
- Near real-time response: Fast response of a sensor is always desirable. Near real-time response is another significant feature of the present sensor. The sharp decrease in the ringdown time in Figure 5 shows that the response time was 1.5 s. Taking the 100 measuring events into consideration, a single measuring time is only 15 milliseconds. In application in civil structure monitoring, this response time has significant socio-economic impact in structure damage mitigation, *i.e.*, in the case of natural disasters.
- High detection sensitivity and large dynamic range: Owing to the high baseline stability, ∼0.33%, this FLRD crack senor potentially has a crack detection sensitivity of tens of microns. As a typical example, the *unit-3* has a detection sensitivity of 31 μm in terms of SCW. On the other hand, crack sensing was successfully carried out for SCW as large as 3.5 mm. Therefore, a large dynamic range of crack detection, from tens of microns to a few mm, can be expected from this sensor. Given the fact that the sensing is accomplished with a bare SMF with simplicity in the construction of sensor, this level of sensitivity and dynamic range for crack detection is still practically appreciable in some applications.

An additional feature of the FLRD crack sensor, which has not been demonstrated in this work, is the networking capability. Due to the time-domain sensing scheme of the FLRD-based sensing [42], the uniform sensing signal, *time*, can be readily multiplexed with the signals from multiple FLRD sensor units, even with different sensing functions, to achieve a large scale sensing network. Certainly, current FLRD crack sensors have their own limitations. For instance, at this stage, the FLRD crack sensors can only monitor sensing events, and cannot pinpoint a crack location and measure the crack-width. Secondly, in order to achieve distributed sensing, multiple sensor heads (units) need to be assembled in a sensor system to detect crack locations and as well as time sequence of a series of cracking events when these occur. All of these are topics that remain unaddressed.

## 4. Conclusions

A new type of FLRD-based sensors for crack detection in concrete structures has been developed. The sensing principle and instrumentation is described. A bare single mode fiber, without any modification and treatment, was shown capable of detecting surface cracks with a theoretical detection sensitivity of microns (μm). Performance of the sensors was tested with actual concrete bars made in our laboratory. Responses of the sensors toward the manually produced cracks on the surface of concrete bars were recorded. The sensors exhibited a fast response (∼1.5 s) to the cracking events. In this exploratory study, the surface crack width (SCW) was detected with a theoretical detection sensitivity of 31 μm. The sensor responded efficiently to a SCW up to 3.5 mm. Therefore, a large dynamic range of crack detection, from microns (μm) to a few millimeters, is expected from this sensor. This is the first time that the FLRD technique has been demonstrated for crack detection in actual concrete structures.
